# Improved Water Barrier Properties of Calcium Alginate Capsules Modified by Silicone Oil

**DOI:** 10.3390/gels2020014

**Published:** 2016-04-07

**Authors:** Brian G. Zukas, Nivedita R. Gupta

**Affiliations:** Department of Chemical Engineering, University of New Hampshire, Durham, NH 03824, USA; bgf7@unh.edu

**Keywords:** calcium alginate, inverse gelation, emulsion, encapsulation, water barrier

## Abstract

Calcium alginate films generally offer poor diffusion resistance to water. In this study, we present a technique for encapsulating aqueous drops in a modified calcium alginate membrane made from an emulsion of silicone oil and aqueous alginate solution and explore its effect on the loss of water from the capsule cores. The capsule membrane storage modulus increases as the initial concentration of oil in the emulsion is increased. The water barrier properties of the fabricated capsules were determined by observing the mass loss of capsules in a controlled environment. It was found that capsules made with emulsions containing 50 wt% silicone oil were robust while taking at least twice the time to dry completely as compared to capsules made from only an aqueous alginate solution. The size of the oil droplets in the emulsion also has an effect on the water barrier properties of the fabricated capsules. This study demonstrates a facile method of producing aqueous core alginate capsules with a modified membrane that improves the diffusion resistance to water and can have a wide range of applications.

## 1. Introduction

The use of encapsulation can be seen in cosmetics, food products, drugs/vitamins and even self healing materials. The wide range of industries that use encapsulation techniques underscores its importance in the manufacture of modern products [1,2,3,4,5]. Out of the many materials used for encapsulation purpuses, alginate is an attractive material due to the simplicity of the encapsulation reaction, the biocompatibility of alginate [6], and its ability to be modified or combined with other bio-materials [7]. Generally, commercial methods of encapsulation break down into two groups, encapsulation through mechanical methods and encapsulation through chemical methods [8]. It is well established that alginate molecules can be readily crosslinked into a biopolymer by a variety of divalent ionic species [9]. Many techniques have been established to form alginate capsules using the rapid and facile crosslinking of this material in order to form capsules in a wide range of sizes. Capsules containing both aqueous and oil cores have been formed from compound drops where an external drop of alginate solution surrounds a liquid core. The capsule is then formed by crosslinking the external alginate coating by a bath [10] or a flowing solution containing crosslinking ions [11]. Variations on this method include introducing drops of an ionic solution into a volume of alginate solution [12] or dispersing calcium carbonate particles into an aqueous layer of alginate solution [13].

The rate of controlled release from alginate capsules, beads, and films has been an area of much focus. Solid fillers, such as starch, clays and other inorganic materials, have been used to modify the diffusion of compounds through alginate [14,15,16]. Incorporating magnetic nanoparticles into the alginate matrix of crosslinked beads has also been seen to decrease the drying rate of the beads [17]. Coatings of alginate beads with materials such as poly(methyl methacrylate) has been successfully demonstrated as well [18]. Alginate films offer good resistance to oxygen diffusion but not to water as crosslinked alginate is a hydrogel [19]. Slowing the rate of diffusion for molecules such as water is difficult due to the small size of the molecules [20]. Low concentrations of lipids have been incorporated into alginate films in order to improve the film’s barrier properties [19,21]. High concentrations of various oils, up to 85 wt%, have been incorporated into the core of the capsules [22,23,24]. 

However, these studies were focused on the inclusion of the oil as the compound of interest. In this work, we present a technique to incorporate high concentrations of silicone oil into the membrane of a calcium alginate capsule in order to prevent the loss of the core liquid. This technique is used to encapsulate cores of an aqueous dextran solution and reduce the permeability of the capsule membrane to water molecules. The effect of changing the initial oil content of the silicone oil-alginate emulsion and the viscosity of silicone oil on the rate of water loss from the capsules is also investigated.

## 2. Results

In the following sections, we compare the membrane, shape and size, and drying properties of the capsules synthesized by varying the initial oil content, mixing technique, or oil viscosity in the emulsion gelling bath. There is no easy way to quantify the amount of oil actually trapped in the membrane during the cross-linking process. Hence, the legends in the figures in the following sections are meant to imply the initial oil contents in the gelling bath and not the actual weight percent of oil in the capsule membrane. 

### 2.1. Membrane Rheological Behavior

**Figure 1:**
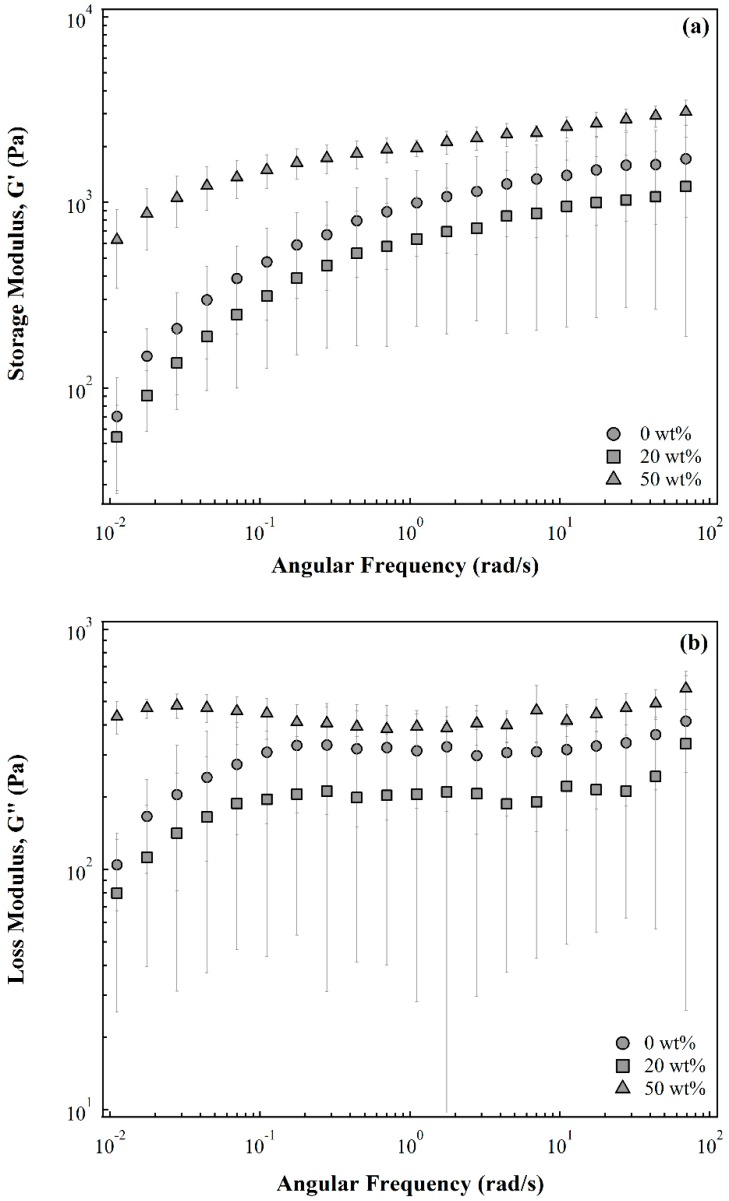
(**a**) Storage modulus, G’ and (**b**) loss modulus, G’’ as a function of angular frequency for alginate emulsion gels prepared from emulsions with different initial silicone oil compositions.

We begin by studying the rheological properties of the capsule membrane as the presence of silicone oil in the gelling bath during the cross-linking process can modify the mechanical properties of the membrane formed, which can impact processing of the capsules post synthesis. The storage and loss moduli, $G^{\prime }$ and $G^{\prime \prime }$, as a function of angular frequency for the membranes formed from pure alginate solution, 20 wt%, and 50 wt% silicone oil-alginate solution emulsions are shown in Figure 1. As the silicone oil concentration in the emulsion increases from 0 wt% to 20 wt%, the storage or loss moduli do not change significantly. The hydrogel made from a 50 wt% silicone oil-alginate solution emulsion not only shows a higher loss modulus but also has a higher storage modulus as compared to hydrogels made from the 0 wt% or 20 wt% silicone oil emulsion. For 50 wt% silicone oil-alginate solution emulsion, there is less alginate available to be crosslinked by the same concentration of Ca^2+^ ions. A higher degree of crosslinking for the hydrogel made from 50 wt% silicone oil-alginate solution emulsion results in a stiffer membrane. These results confirm that silicone oil does get incorporated into the membrane during the cross-linking process and affects the mechanical properties of the membrane. It should be noted that the rheological measurements were made on a flat membrane synthesized using a process very similar to the one used to synthesize the capsules. While we expect the storage and loss moduli for the membranes in the capsules to differ to some extent from those reported in Figure 1, we expect the trends seen with varying oil contents to still apply.

### 2.2. Capsule Properties

**Figure 2:**
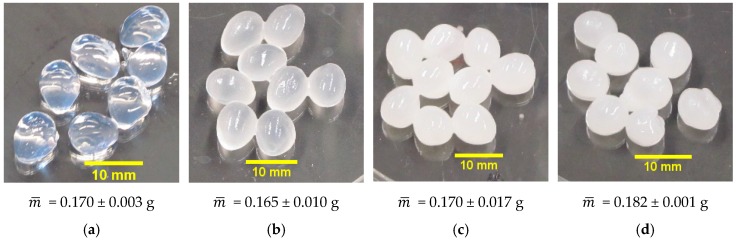
Images and the average mass per capsule,$\;\bar{m}$, of alginate capsules made from (**a**) pure alginate solution, (**b**) 20 wt% silicone oil-alginate emulsion, (**c**) 50 wt% silicone oil-alginate emulsion, and (**d**) 60 wt% silicone oil-alginate emulsion.

Figure 2 shows images of capsules fabricated by changing the oil content from 0 wt% to 60 wt% silicone oil in the gelling bath. The composition of the emulsion used to produce a batch of capsules influences the amount of oil that will be incorporated into the capsule membrane. Increasing the oil content in the capsule membrane increases the opacity of the membranes formed compared to the relatively translucent capsules made from pure alginate solution. Figure 2 also shows the average mass of each capsule, $\bar{m}$, obtained by measuring the initial mass of 10 capsules used for the drying experiments. The reported average and standard deviation value corresponds to the average of three samples of 10 capsules each. The average mass of capsules made from pure alginate and 20 wt% silicone oil-alginate solution emulsions is slightly lower than the masses of capsules made with 50 wt% and 60 wt% silicone oil-alginate solution emulsions. This difference is assumed to have a negligible effect on the analysis of the results. 

It has been previously established that there are many parameters that affect the shape of an alginate capsule. The size of the core drop, the height that the core drop falls from, the viscosities of the drop and the gelling bath, and the stirring rate are parameters known to play a role in determining capsule shape [23,25]. For the experimental setup used in this work to produce capsules, the liquid core drops are first formed at the tip of a needle and then dripped into the emulsion gelling bath. As we change the oil content in the emulsion, several properties of the gelling bath such as density and interfacial tension change to a limited extent. The overall viscosity of the gelling bath, however, changes significantly and is expected to have the greatest impact on the shape of the capsules synthesized. The dispersed phase of the gelling bath, silicone oil, is a Newtonian fluid while the continuous phase, 0.5 wt% alginate solution, exhibits shear thinning behavior. The viscosity versus shear rate behavior of the silicone oil-alginate solution emulsions could not be accurately characterized due to phase separation during the attempted measurements. However, silicone oil and water emulsions have been previously studied and were demonstrated to have significant shear thinning behavior. The low shear rate behavior of the viscosity for a given emulsion was seen to produce viscosity values higher than the individual viscosity of either the oil used or water [26]. As the core drop enters the gelling bath, the shear on the drop from the stirred bath affects the resulting capsule shape. The shear on the drop is dependent both on the velocity of the gelling bath around the drop and the viscosity of the gelling bath. Broadly speaking, the Reynolds number of the gelling bath around the core drop affects the resulting capsule shape. 

**Figure 3:**
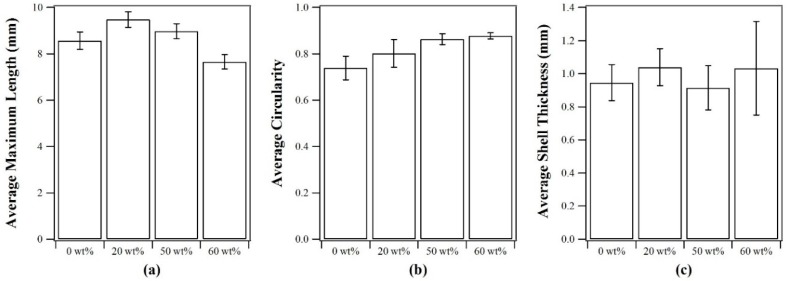
(**a**) Average maximum capsule length, (**b**) average capsule circularity, and (**c**) average shell thickness for capsules prepared from emulsions with different initial silicone oil compositions. Error bars represent standard deviation, *n* = 9.

When fabricating capsules with different oil contents, the core solution was dispensed at the same flow rate and the gelling bath was stirred at a constant speed except for the case of the pure alginate gelling bath. The pure alginate solution could not be stirred at the same rate as the viscosity of the pure alginate solution was lower than the silicone oil-alginate solution emulsions. When the pure alginate gelling bath was stirred at the same rate as the emulsion gelling baths a deep vortex was formed and the increased shear on the core drop produced highly deformed capsules. The 20 wt% silicone oil-alginate solution emulsion had a lower viscosity than both the 50 and 60 wt% silicone oil-alginate solution emulsions, and therefore had a higher Reynolds number at the same set stirring speed. The effect of decreasing Reynolds number at higher silicone oil concentration in the emulsion gelling bath resulted in capsules that were more spherical with shorter maximum lengths, as seen in Figure 3. While the average length and circularity of the capsules varied, the average capsule mass was approximately the same for all concentrations of silicone oil-alginate solution emulsions considered. The initial mass of the capsules was therefore used to normalize the fractional mass loss in the drying experiments. The average shell thickness also did not differ significantly for the capsules made with gelling baths of different initial silicone oil contents, as seen in Figure 3c. 

### 2.3. Capsule Drying

**Figure 4:**
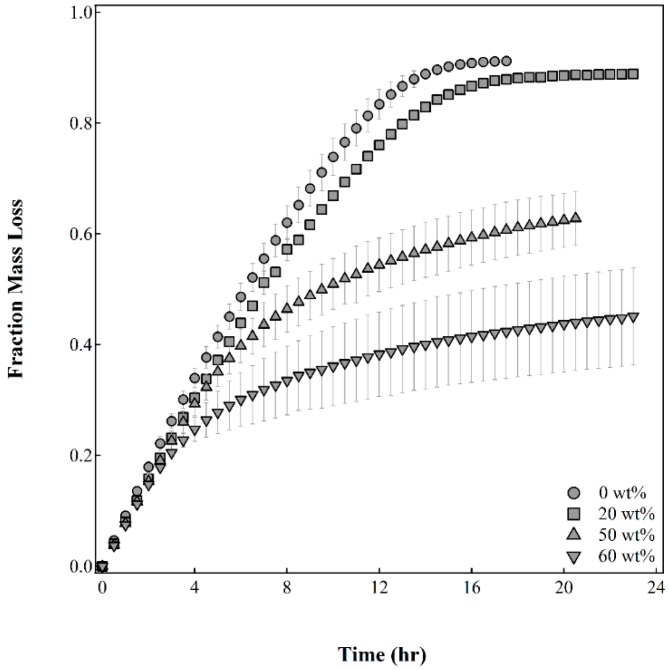
Fraction mass loss as a function of time for capsules made from emulsions with varying initial compositions of silicone oil. Error bars represent standard deviation, *n* = 10.

Figure 4 shows the fraction mass loss as a function of time for capsules fabricated with varying initial oil contents in the gelling bath. Since water is the only fluid in the capsule that can evaporate at room temperature and pressure, the dried capsule will be composed of oil, capsule solids, and dextran in the core. A capsule made from pure alginate solution contains 9 wt% oil and solids while a capsule made from an emulsion with 20 wt% silicone oil increases the oil and solids content to 11 wt%. This is seen as the final fraction mass loss for the two sets of capsules that dried completely in less than 20 h. Since the amount of dextran in each of the capsules fabricated is the same, increasing the oil content of the emulsion increased the amount of oil and capsule solids in the membrane. The capsules made with emulsions containing 50 wt% and 60 wt% silicone oil have a higher content of solids and oil and will plateau at a lower fraction mass loss when dried completely. Since the shell thickness is nearly similar for the different capsule membranes, we can infer that the concentration of oil in the membrane significantly affects the drying behavior of the capsules. 

The fraction mass loss for all four capsule compositions is essentially the same for times up to approximately two hours. This initial slope, before the inflection point in the curves, for all the capsules may represent the loss of moisture on or near the surface of the capsule. As the capsules dry further, the internal water of the capsule has to diffuse through a more tortuous path created by the presence of oil droplets in the capsule membrane. Additionally, as the capsules dry, the oil was observed exuding from the capsules. The exuding oil formed a film on the exterior of the capsule, further decreasing the rate of diffusion. Both of these mechanisms have been observed in the evaporation of water from silicone oil in water emulsions as well [27]. The capsules made from pure alginate solution and 20 wt% silicone oil-alginate solution emulsion continue to dry at a rate similar to the initial drying rate and are completely dry in 15 h and 17 h, respectively. For the capsules made with emulsions containing 50 wt% and 60 wt% silicone oil, inflection points are seen in the fraction mass loss curves at approximately 6 h and 4 h, respectively, where the rate of drying reduces significantly as compared to the initial drying rate.

While data was not continuously collected beyond 24 h, it took more than 35 h for capsules made with emulsions containing 50 wt% and 60 wt% silicone oil to dry completely. The presence of larger amounts of oil in the oil-alginate emulsion improved the water barrier properties of the calcium alginate membrane. Figure 4 shows a large spread in the fraction mass loss data for capsules made with emulsions containing 60 wt% silicone oil suggesting that larger oil contents may also result in compromising the integrity of the membrane causing the larger batch to batch variability. Based on our data, capsules made with emulsions containing 50 wt% silicone oil provide the best water barrier properties while maintaining a robust membrane around the core.

#### Effect of Process Parameters on Capsule Drying

**Figure 5:**
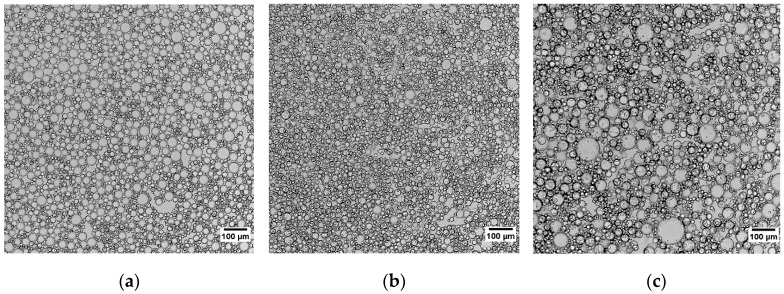
Images of silicone oil-alginate emulsions made with (**a**) 10 cSt silicone oil agitated with magnetic stirrer, (**b**) 10 cSt silicone oil agitated with mechanical stirrer, and (**c**) 500 cSt silicone oil agitated with magnetic stirrer.

**Figure 6:**
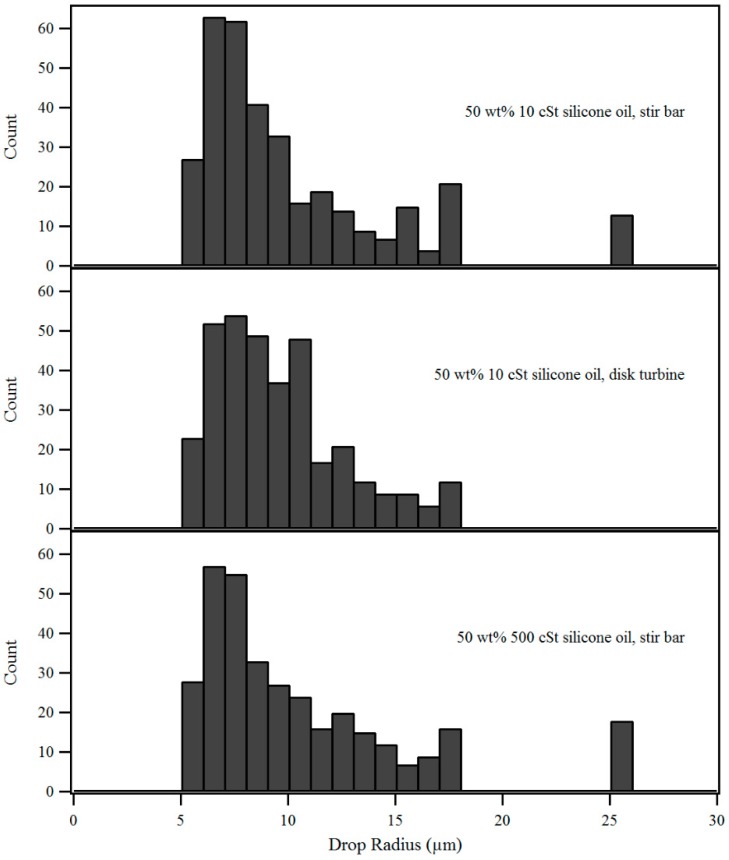
Droplet size distributions for three different emulsion preparations, *n* = 350 for each distribution.

To study how the oil droplet size distribution in the gelling bath emulsion affects the rate of capsule drying, the agitation method for making the emulsion and the viscosity of the silicone oil was varied for a set emulsion composition of 50 wt% silicone oil in alginate solution. Figure 5 shows the images of the emulsions obtained by using 10 cSt silicone oil with a magnetic stir bar and a disk turbine, as well as emulsion formed by using 500 cSt silicone oil with a magnetic stir bar. Figure 6 shows the histograms for the droplet size distribution in the three different emulsions and the median drop size and the inter-quantile range (IQR) of the distributions are seen in Table 1. The median drop size for an emulsion produced with 10 cSt silicone oil but stirred with either a magnetic stir bar and a disk turbine is very similar. The emulsion produced with the turbine has a smaller IQR, indicating a narrower droplet size distribution.

**Figure 7:**
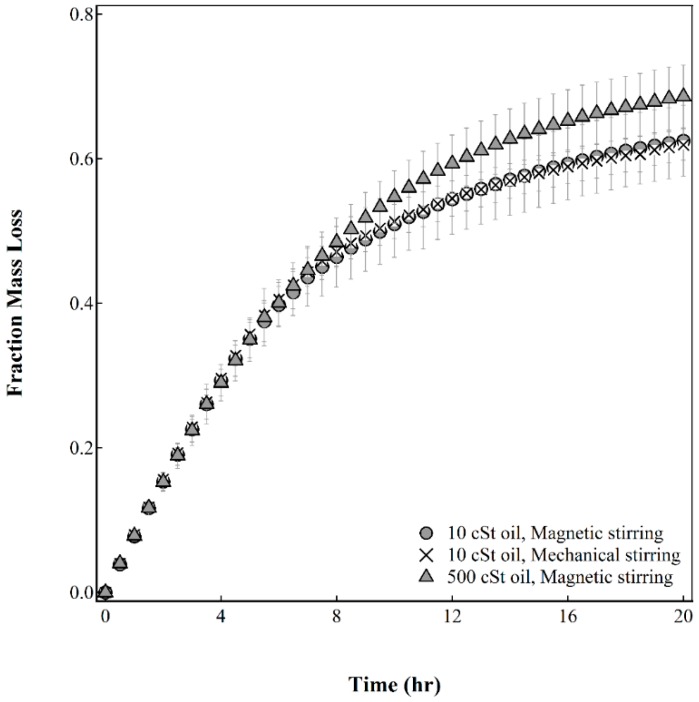
Effect of agitation method and oil viscosity on the fraction mass loss as a function of time. Error bars represent standard deviation, *n* = 10.

However, the resulting capsule drying behavior in Figure 7 does not appear to be sensitive to the spread of the droplet size distribution. The 50 wt% 500 cSt silicone oil emulsion contained a higher median droplet size than the emulsions prepared from 10 cSt silicone oil. The capsules fabricated from the 500 cSt silicone oil emulsion show a higher mass loss than capsules fabricated from the 10 cSt silicone oil emulsions. This indicates that the presence of larger droplets in the emulsion may have a detrimental effect on the inclusion of droplets into the alginate membrane, affecting the water barrier properties of the capsules.

## 3. Conclusions 

In this study, an encapsulation technique is presented to fabricate calcium alginate based capsules from emulsions of silicone oil and alginate solution using the inverse gelation method. The initial oil fraction of the emulsion and the viscosity of the oil affect the fraction of oil trapped in the crosslinked capsule membrane and hence the water barrier properties of the membrane. Increasing the oil content in the oil-alginate emulsion made the capsules more translucent and circular but had a small effect on the initial mass of the capsules. Larger concentrations of oil in the capsule membrane created a more tortuous path for the diffusion of water molecules resulting in an increase in the drying time for the capsule. We found that starting with an oil-alginate emulsion containing 50 wt% silicone oil increased the drying time from 15 h to greater than 35 h and produced a robust capsule membrane. When the viscosity of the silicone oil was increased, the median droplet size of the emulsion was higher which reduced the water barrier properties of the oil-alginate emulsion membrane. The encapsulation technique presented here can be used with any type of oil including plant based oils [28] and provides a facile method of modifying the properties of a calcium alginate capsule membrane that improves its water barrier properties.

## 4. Materials and Methods 

### 4.1. Solution and Emulsion Preparation

The capsule core solution consisted of 5 wt% calcium chloride dihydrate (Fisher Scientific, Waltham, MA, USA) and 10 wt% dextran (average MW. 500,000 g·mol^−1^, Spectrum Chemical, New Brunswick, NJ, USA) dissolved in ultra-pure (Milli-Q, 18.2 MΩ·cm, Billerica, MA, USA) water. The gelling bath consisted of either 0.5 wt% aqueous alginate solution or a silicone oil-alginate solution emulsion. The 0.5 wt% alginate solution was prepared by adding alginic acid, sodium salt (MW: 450,000–550,000 g·mol^−1^, M/G ratio 1.5–1.6, Acros Organics, Morris Plains, NJ, USA) to ultra-pure water. The silicone oil-alginate solution emulsions were made by mixing the 0.5 wt% alginate solution with silicone oil (Clearco Products, Willow Grove, PA, USA, 10 cSt or 500 cSt) to reach the desired mass fraction of oil in the emulsion for a total emulsion weight of 60 g. The viscosities of the alginate solution and the silicone oils are presented in Table 2. 0.033 g of the surfactant Triton X-100 (Alfa-Aesar, Ward Hill, MA, USA) was added to help stabilize the emulsion. The emulsions were stirred by a magnetic stir bar at 1000 rpm for two days to ensure consistency of emulsion formation amongst various batches. A trial was also conducted using an overhead mechanical stirrer (Lightnin L1U08F with a 2 in. diameter, 6 blade disk turbine, Rochester, NY, USA) at 1200 rpm for one hour.

### 4.2. Capsule Fabrication

The capsule core solution of calcium chloride and dextran was pumped at 1 mL·min^−1^, using a syringe pump, to a 14 gauge blunt tip dispensing needle. Drops of the core solution were dripped from the tip of the needle into the gelling bath consisting of either alginate solution or the silicone oil-alginate emulsion [12]. Once the desired number of core drops were dispensed, the membrane formation reaction step was allowed to proceed for 10 min under ambient conditions. The reaction mixture was continuously stirred by a magnetic stir bar during the drop formation and reaction step. The capsules were then removed from the reactor and placed on a shaker table in a 5 wt% calcium chloride solution for 10 min to complete the cross-linking process. Following that, the capsules were removed from the calcium chloride solution and placed in deionized water for one minute to help remove residual calcium chloride solution from the surface of the capsules. The capsules were then removed from the water and lightly dabbed using kimwipes to remove any surface water films from the capsule membrane surface. The capsules were immediately used for capsule size or drying measurements.

### 4.3. Capsule Size Measurements

Images of capsules and capsule shell were taken with a CMOS camera (Pixelink PL-B741U, Ottawa, ON, Canada, 1.3 MP resolution) perpendicular to the benchtop. The images were then analyzed using ImageJ particle analysis tool to determine the maximum length and circularity of the capsules [29].

### 4.4. Rheological Measurements

The viscosity of the core solution, alginate solution, and silicone oils were measured on a TA Instruments AR-550 Advanced Rheometer (New Castle, DE, USA) using a 40 mm, 2° cone geometry. The temperature for these experiments was maintained at 25 °C by the rheometer’s built in Peltier plate. The viscosities of the silicone oil-alginate solution emulsions could not be successfully measured due to significant phase separation that occurred during the viscosity measurements. 

The storage and loss moduli were also measured for the silicone oil-alginate hydrogel membranes fabricated from emulsions with differing silicone oil mass fractions. A hydrogel membrane disk was first fabricated by cutting a lab napkin into a 40 mm diameter circle. The napkin was then soaked in the same core solution that was used to fabricate the capsules [30]. The wet napkin was then place in a beaker containing 60 grams of either alginate solution or a silicone oil-alginate solution emulsion and the same membrane fabrication procedure described earlier was followed. Once a membrane was formed around the napkin the membrane was cut away from the napkin using an exacto knife. The rheological properties of the hydrogel membrane disk were measured using the AR-550 Advanced Rheometer with a 40 mm parallel plate geometry at a height of 800 μm. Excess hydrogel was cut away with an exacto knife and any water that had exuded was wiped away. The linear viscoelastic region of the hydrogel was found using an oscillatory stress sweep at 25 °C. The results from the oscillatory stress sweep were then used to choose the value of the oscillatory stress for the frequency sweep experiment.

### 4.5. Drying Measurements

The moisture barrier properties of the membrane was evaluated by measuring the fraction mass loss of the capsules as a function of time. For each drying trial, ten capsules on a petri dish were placed on a digital scale (Mettler Toledo, Columbus, OH, USA, 1 mg readability). The digital scale along with the petri dish was kept in a chamber where the humidity was maintained at 38 ± 2% using a digital humidity controller (Willhi, Shenzhen, China) . Humid air was supplied to the chamber using a cool mist humidifier (Sunbeam, Boca Raton, FL, USA) while dry air was continuously supplied at a low flow rate from the laboratory’s compressed air line. The temperature of the container was not controlled but remained at 20 ± 1 °C. The total mass of the ten capsules and the humidity and temperature inside the chamber were recorded every 30 min for 20 h or until the capsules dried completely. The fraction mass loss was calculated as:

(1) $$\text{Fraction Mass Loss}=\frac{m_{i}-m_{t}}{m_{i}}$$

where $m_{i}$ represents the initial mass of the ten capsules at *t =* 0 and $m_{t}$ represents the mass of the ten capsules at any time, *t*. The experiments were repeated with three separate batches of ten capsules each.

### 4.6. Emulsion Droplet Size Distribution Analysis

The droplet size distributions were measured for the silicone oil-alginate solution emulsions prepared from: 10 cSt silicone oil stirred by a magnetic stir bar, 10 cSt silicone oil stirred by an overhead disk turbine and a 500 cSt silicone oil stirred by a magnetic stir bar. Samples from the emulsions were diluted by a 0.5 wt% alginate solution with TX-100 in order to properly visualize individual drops. Images were recorded on an inverted microscope (Nikon TE-200, Melville, NY, USA) using an attached CMOS camera (MotionPro X3, Tallahassee, FL, USA). The images were then analyzed using the ImageJ particle analysis tool.

## Figures and Tables

**Table 1:** Description statistics for the droplet size distributions of three different emulsion preparations, *n* = 350 for each.

Emulsion	Median Radius (µm)	IQR (µm)	Mean (µm)	Std. Dev. (µm)
50 wt% 10 cSt, stir bar	8.52	5.14	10.3	5.02
50 wt% 10 cSt, disk turbine	8.91	3.82	9.57	3.04
50 wt% 500 cSt, stir bar	9.11	6.04	11.3	6.34

**Table 2:** Viscosity of various solutions used in the fabrication of modified alginate capsules. Data was measured at 25 °C.

Solution	Viscosity (mPa.s)
10% Dextran Solution	33 ± 3
0.5 wt% Alginate Solution	30.7 ± 0.1 (zero shear rate)
10 cSt Silicone Oil	9.7 ± 0.1
500 cSt Silicone Oil	505 ± 4

## References

[1] Datta S.S., Abbaspourrad A., Amstad E., Fan J., Kim S.-H., Romanowsky M., Shum H.C., Sun B., Utada A.S., Windbergs M. (2014). 25th Anniversary Article: Double Emulsion Templated Solid Microcapsules: Mechanics And Controlled Release. Adv. Mater..

[2] Desai K.G.H., Park H.J. (2005). Recent Developments in Microencapsulation of Food Ingredients. Dry. Technol. Int. J..

[3] Gonnet M., Lethuaut L., Boury F. (2010). New trends in encapsulation of liposoluble vitamins. J. Controlled Release.

[4] Kim C.A., Joung M.J., Ahn S.D., Kim G.H., Kang S.-Y., You I.-K., Oh J., Myoung H.J., Baek K.H., Suh K.S. (2005). Microcapsules as an electronic ink to fabricate color electrophoretic displays. Synth. Met..

[5] Samadzadeh M., Boura S.H., Peikari M., Kasiriha S.M., Ashrafi A. (2010). A review on self-healing coatings based on micro/nanocapsules. Prog. Org. Coat..

[6] Gasperini L., Mano J.F., Reis R.L. (2014). Natural polymers for the microencapsulation of cells. J. R. Soc. Interface.

[7] Broderick E., Lyons H., Pembroke T., Byrne H., Murray B., Hall M. (2006). The characterisation of a novel, covalently modified, amphiphilic alginate derivative, which retains gelling and non-toxic properties. J. Colloid Interface Sci..

[8] Singh M.N., Hemant K.S.Y., Ram M., Shivakumar H.G. (2010). Microencapsulation: A promising technique for controlled drug delivery. Res. Pharm. Sci..

[9] Mørch Ý.A., Donati I., Strand B.L. (2006). Effect of Ca^2+^, Ba^2+^, and Sr^2+^ on Alginate Microbeads. Biomacromolecules.

[10] Bremond N., Santanach-Carreras E., Chu L.-Y., Bibette J. (2010). Formation of liquid-core capsules having a thin hydrogel membrane: Liquid pearls. Soft Matter.

[11] Kim C., Chung S., Kim Y.E., Lee K.S., Lee S.H., Oh K.W., Kang J.Y. (2011). Generation of core-shell microcapsules with three-dimensional focusing device for efficient formation of cell spheroid. Lab Chip.

[12] Nigam S.C., Tsao I.-F., Sakoda A., Wang H.Y. (1988). Techniques for preparing hydrogel membrane capsules. Biotechnol. Tech..

[13] Liu L., Wu F., Ju X.-J., Xie R., Wang W., Niu C.H., Chu L.-Y. (2013). Preparation of monodisperse calcium alginate microcapsules via internal gelation in microfluidic-generated double emulsions. J. Colloid Interface Sci..

[14] Gal A., Nussinovitch A. (2007). Hydrocolloid carriers with filler inclusion for diltiazem hydrochloride release. J. Pharm. Sci..

[15] López-Córdoba A., Deladino L., Martino M. (2014). Release of yerba mate antioxidants from corn starch–alginate capsules as affected by structure. Carbohydr. Polym..

[16] Vale J., Justice R., Schaefer D., Mark J. (2005). Calcium Alginate Barrier Films Modified by Montmorillonite Clay. J. Macromol. Sci. B Phys..

[17] Degen P., Leick S., Siedenbiedel F., Rehage H. (2012). Magnetic switchable alginate beads. Colloid Polym. Sci..

[18] Wah Chan L., Liu X., Heng P.W.S. (2005). Liquid phase coating to produce controlled-release alginate microspheres. J. Microencapsul..

[19] Tapia M.S., Rojas-Graü M.A., Rodríguez F.J., Ramírez J., Carmona A., Martin-Belloso O. (2007). Alginate- and Gellan-Based Edible Films for Probiotic Coatings on Fresh-Cut Fruits. J. Food Sci..

[20] Patchan M.W., Baird L.M., Rhim Y.-R., LaBarre E.D., Maisano A.J., Deacon R.M., Xia Z., Benkoski J.J. (2012). Liquid-Filled Metal Microcapsules. ACS Appl. Mater. Interfaces.

[21] Hambleton A., Debeaufort F., Bonnotte A., Voilley A. (2009). Influence of alginate emulsion-based films structure on its barrier properties and on the protection of microencapsulated aroma compound. Food Hydrocoll..

[22] Chan E.-S. (2011). Preparation of Ca-alginate beads containing high oil content: Influence of process variables on encapsulation efficiency and bead properties. Carbohydr. Polym..

[23] Abang S., Chan E.-S., Poncelet D. (2012). Effects of process variables on the encapsulation of oil in ca-alginate capsules using an inverse gelation technique. J. Microencapsul..

[24] Martins E., Renard D., Davy J., Marquis M., Poncelet D. (2015). Oil core microcapsules by inverse gelation technique. J. Microencapsul..

[25] Chan E.-S., Lee B.-B., Ravindra P., Poncelet D. (2009). Prediction models for shape and size of ca-alginate macrobeads produced through extrusion–dripping method. J. Colloid Interface Sci..

[26] Saadevandi B.A., Zakin J.L. (1996). A Study of Silicone Oil-in-Water Emulsions. Chem. Eng. Commun..

[27] Aranberri I., Binks B.P., Clint J.H., Fletcher P.D.I. (2004). Evaporation rates of water from concentrated oil-in-water emulsions. Langmuir.

[28] Zukas B.G. (2014). Encapsulation of Anti-Traction Material. Msc Thesis.

[29] Schneider C.A., Rasband W.S., Eliceiri K.W. (2012). NIH Image to Image J: 25 Years of image analysis. Nat. Methods.

[30] Bron J.L., Vonk L.A., Smit T.H., Koenderink G.H. (2011). Engineering alginate for intervertebral disc repair. J. Mech. Behav. Biomed. Mater..

